# Revisiting the Role of NAG across the Continuum of Kidney Disease

**DOI:** 10.3390/bioengineering10040444

**Published:** 2023-04-04

**Authors:** Ruder Novak, Grgur Salai, Stela Hrkac, Ivana Kovacevic Vojtusek, Lovorka Grgurevic

**Affiliations:** 1 Center for Translational and Clinical Research, Department of Proteomics, School of Medicine, University of Zagreb, 10000 Zagreb, Croatia; 2 Department of Pulmonology, University Hospital Dubrava, 10000 Zagreb, Croatia; 3 Department of of Clinical Immunology, Allergology and Rheumatology, University Hospital Dubrava, 10000 Zagreb, Croatia; 4 Department of Nephrology, Arterial Hypertension, Dialysis and Transplantation, University Hospital Center Zagreb, 10000 Zagreb, Croatia; 5 Department of Anatomy, “Drago Perovic”, School of Medicine, University of Zagreb, 10000 Zagreb, Croatia

**Keywords:** N-acetyl-beta-D-glucosaminidase, NAG, chronic kidney disease, acute kidney injury, biomarker

## Abstract

Acute and chronic kidney diseases are an evolving continuum for which reliable biomarkers of early disease are lacking. The potential use of glycosidases, enzymes involved in carbohydrate metabolism, in kidney disease detection has been under investigation since the 1960s. N-acetyl-beta-D-glucosaminidase (NAG) is a glycosidase commonly found in proximal tubule epithelial cells (PTECs). Due to its large molecular weight, plasma-soluble NAG cannot pass the glomerular filtration barrier; thus, increased urinary concentration of NAG (uNAG) may suggest injury to the proximal tubule. As the PTECs are the workhorses of the kidney that perform much of the filtration and reabsorption, they are a common starting point in acute and chronic kidney disease. NAG has previously been researched, and it is widely used as a valuable biomarker in both acute and chronic kidney disease, as well as in patients suffering from diabetes mellitus, heart failure, and other chronic diseases leading to kidney failure. Here, we present an overview of the research pertaining to uNAG’s biomarker potential across the spectrum of kidney disease, with an additional emphasis on environmental nephrotoxic substance exposure. In spite of a large body of evidence strongly suggesting connections between uNAG levels and multiple kidney pathologies, focused clinical validation tests and knowledge on underlining molecular mechanisms are largely lacking.

## 1. Introduction

The kidney is a vital organ with critical functions in maintaining body fluid balance, waste product secretion, hormone production, and acid-base and electrolyte homeostasis [1]. It makes up less than 1% of the total body mass and receives roughly a quarter of the cardiac blood output, making it susceptible to many toxic agents [2]. The workhorse of the kidney is the proximal tubule epithelial cell (PTEC) system, which performs much of the filtration and reabsorption, hence its role as a major starting point in both acute and chronic disease scenarios [3,4,5,6,7]. As renal tissue is stressed, multiple underlying mechanisms and molecular pathways can induce acute kidney damage that can, over time, lead to chronic kidney disease (CKD)—a worsening glomerular, tubular and/or vascular impairment [8]. Briefly, noxious stimuli cause injury to PTECs, which stimulate inflammation, recruit myofibroblasts, and produce a plethora of profibrogenic molecules that drive interstitial inflammation and fibrosis. Furthermore, uninjured tubular cells face an increased tubular transport workload, which leads to hypoxia, anaerobic metabolism utilization, and acidosis [6,9]. Renal tissue has limited regeneration capabilities, so the ensuing vicious cycle of damage-induced inflammation sustains fibrosis, which over time deteriorates tissue function, leading to end-stage renal disease and the need for kidney transplantation or dialysis [10].

Diagnosis of kidney disease currently relies on the estimation of functional parameters, such as glomerular filtration rate (GFR), kidney biopsies, or the more sensitive indicators such as detection of proteinuria or assessment of urinary enzymes [11]. The diverse etiology of CKD impedes our ability to predict the dynamics of its progression, its long-term outcomes, and adequate therapeutic approaches. As this global public health problem is not yet fully understood on the molecular level, many potential disease biomarkers have so far been extensively reviewed as possible early predictors of disease progression. The problem with using plasma biomarkers is that they usually reflect systemic endothelial dysfunction or inflammation; i.e., they are not specific biomarkers of kidney damage [12]. On the contrary, urinary biomarkers of tubular dysfunction show more promise in that regard. Recent efforts have focused on recognizing early tubular damage, that is, prior to PTEC injury and the appearance of any clinical and functional signs [13,14]. In that regard, the prediction of acute kidney injury (AKI) in patients at risk by the combined detection of urinary “acute kidney stress” molecules tissue inhibitor of metalloprotease-2 (TIMP-2) and insulin-like growth factor-binding protein 7 (IGFBP7) was recently approved by the U.S. Food and Drug Administration [15,16]. The onset of tissue damage causes urinary secretion of further mediators that are traditionally divided into three groups: (a) PTEC enzymes (N-acetyl-beta-D-glucosaminidase (NAG), α-glutathione S-transferase, and γ-glutamyl transpeptidase-GGT), whose concentrations in urine rise as a result of stress-induced metabolic changes; (b) molecules that are specifically upregulated upon PTEC damage; and (c) urinary low-molecular-weight proteins, such as alpha-1 and beta-2 microglobulin, that are secreted elsewhere in the body, filtrated by the glomeruli, reabsorbed completely by the PTECs, and found in urine only in states of tubular dysfunction [17,18]. A promising early biomarker of tubular damage is the neutrophil gelatinase-associated lipocalin (NGAL) that can potentially differentiate between reversible and irreversible injury, with important implications in customizing the therapeutic approach [19]. Other molecules studied in this regard include the kidney injury molecule-1 (KIM-1), the liver-type fatty acid-binding protein (L-FABP), and interleukin-18 (IL-18); however, evidence from multiple clinical studies shows a variable potential to predict the initiation and/or progression of kidney injury [20,21].

In contrast to other tubular cell enzymes, the PTEC lysosomal enzyme NAG has been extensively studied and has been shown to be a sensitive, persistent, and robust indicator of kidney injury. The amount of NAG in urine can be related to tubular damage and can be rapidly quantified using reproducible and well-defined enzymatic assays [22]. The first investigations regarding NAG in kidney disease date back over 50 years, as Dance et al. proposed in 1969 that glycosidases might be used as biomarkers of kidney damage [23]. The role of urinary NAG (uNAG) concentration as a potential marker of tubular damage was further solidified by Lockwood and Bosmann in 1979, who found that nephrotoxic doses of aspirin led to its dose-dependent increase [24,25,26]. Since uNAG is elevated in multiple conditions associated with kidney injury, as well as in conditions resulting from exposure to nephrotoxic substances, there is a need to uncover the pathophysiological mechanism of this phenomenon. A comprehensively curated review of published studies would to defining the position of uNAG as a biomarker of kidney injury.

## 2. Molecular Biology of NAG

**Figure 1:**
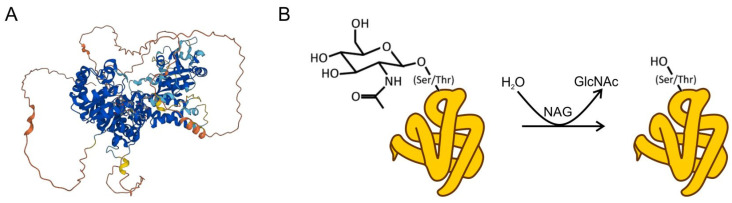
(**A**) Structure of NAG, as predicted by the AlphaFold protein structure database [32]. (**B**) NAG catalyzes the reversible hydrolysis of the attached GlcNAc structure.

NAG is a large protein of 916 amino acids (the median protein size in humans is 375) with a predicted molecular mass of 140 kDa [27]. It is an evolutionally conserved hydrolase that breaks down oligosaccharides, modulating the O-glycosilation of proteins. It acts by cleaving *N*-acetylglucosamine (GlcNAc), a monosaccharide that is reversibly added to proteins at the oxygen atom of serine and threonine side chains (Figure 1). In contrast to other forms of glycosylation that attach to complex branched sugar structures, O-GlcNAc is a small tag that cycles on and off proteins and does not undergo elongation [28]. This post-translational modification is common across the evolutionary tree—it has been found in all metazoans, some prokaryotes, and viruses. The modification serves as a nutrient and stress sensor, and it regulates all basic biochemical processes examined, rangeing from signalling, transcription, and mitochondrial activity, to cytoskeletal functions [29,30]. Unsurprisingly, given its fundamental roles, it has significant implications in etiopathogenic webs leading to chronic diseases such as diabetes, cancer, and neurodegenerative disease [31].

NAG presents in high concentrations in the PTECs as a lysosomal brush-border enzyme and the most active glycosidase. Thus far, three isoforms of NAG have been recognized in humans, differing in the N-terminal length of the (presumably) catalytic domain of the protein. Although all isoforms retain catalytic activity, it is significantly diminished in the truncated protein [33]. Two of them have been classified: isoenzyme A is primarily located in the soluble part of lysosomes and is excreted by exocytosis and found in urine under physiological conditions, and NAG “B” isoenzyme is predominantly located at the lysosomal membrane and was found to be increased when tubular injury occurred [34,35,36]. However, in spite of an interesting prospect, recent and robust research regarding NAG isoenzymes is lacking.

Due to its large molecular weight, plasma-soluble NAG cannot pass the glomerular filtration barrier [7,37,38]. The enzyme was found to be prone to caspase-3 cleavage, but surprisingly, this had no effect on its activity as an O-GlcNAc-ase. Its activity was found to be inhibited in a dose-dependent manner by diabetogenic agents, such as the toxin alloxan and antibiotic streptozotocin [39,40]. Limited data suggest that NAG is expressed on the gene level in every tissue examined, from skeletal and heart muscle to the lung, liver, pancreas, kidney, and placenta. However, the largest mRNA expression levels were detected in brain tissue [28]. Interestingly, its expression on the protein level has not been extensively studied. According to the Human Atlas, the expression of NAG on the protein level has been confirmed in kidney tubules, but not in glomeruli [41].

Urinary NAG primarily originates from PTECs that, under physiological conditions, excrete small and stable quantities of NAG by exocytosis [35]. When PTECs are injured, an increase in urinary NAG concentration can be observed [37,42]. Furthermore, as NAG is involved in carbohydrate metabolism, it is possible that the exposure of proximal tubule epithelial cells to increased glucose levels leads to increased urinary NAG concentrations [43,44].

## 3. NAG in Kidney Injury

### 3.1. NAG in the Setting of AKI

AKI is a common clinical entity whose main outcome is a rapid decline of renal function [45]. The clinical definition and classification of AKI are provided by the 2012 Kidney Disease: Improving Global Outcomes (KDIGO) clinical practice guidelines, which rely on changes in serum creatinine and urine output. The definition and treatment recommendations remain points of interest since there are limitations of a classification system based on creatinine in catabolic or sarcopenic patients [46,47]. AKI presents a clinical problem commonly faced by a majority of physicians, but it also presents a global healthcare problem, as it has been significantly associated with mortality, length of hospital stay, and healthcare cost. Furthermore, CKD is recognized as a common sequela of AKI. Even in resolved AKI, KDIGO guidelines suggest these patients should be considered as at increased risk of CKD [46].

AKI can originate from multiple causes such as decreased renal perfusion, renal tubule injury from toxins or obstruction, tubulointerstitial inflammation, and primary reduction in the glomerulus’ filtering capacity. The majority of AKI cases are due to ischemia and toxins, which cause loss of integrity and polarity in the kidney epithelium, leading to necrosis and apoptosis. This, in turn, leads to inadequate filtration and filtrate leak, i.e., proteinuria and the possibility of subsequent identification of specific proteins in urine [22]. Even though early recognition of AKI is essential as it could improve patient outcomes, its early diagnosis remains a challenge [48,49]. Kidney injury starts by inducing biological and molecular changes that, over time, evolve into cellular damage [45]. Therefore, the discovery and validation of a reliable biomarker for AKI prediction and early diagnosis seems prudent, as it would allow early diagnosis and inform on the progression of AKI, thereby improving treatment strategies [50].

Among candidate molecules, NAG, more precisely its urine concentration, has been studied extensively [22]. Importantly, its elevated urine levels in kidney injury are an early sign of disease since they precede the elevation of serum creatinine, a sign of worsening renal filtration [51]. NAG has previously been described as one of the promising novel markers of AKI; however, further research was warranted in order for it to be validated, namely its efficacy in different age groups and its utility in the setting of different pathophysiological circumstances (e.g., drug-induced AKI, critically ill patients, etc.) [45]. In recent years, numerous studies addressing these questions have been published and show promising results.

Multiple recent studies have evaluated NAG as a marker of AKI in patients with cardiovascular diseases. In a study group of patients with chest pain, NAG was compared with other markers, such as creatinine and cystatin C, and was shown to be the only marker with a promising potential for AKI prediction. Its additional clinical value was also shown in its possibility to predict the necessity of renal replacement therapy [52]. In addition to acute cardiovascular settings, NAG has also been shown to indicate tubular injury in chronic heart failure [53]. Furthermore, Fujigaki et al. analyzed patients with AKI due to minimal change nephrotic syndrome (MCNS) by using immunohistochemical expression of vimentin as a marker of tubular injury and dedifferentiation. They found proximal convoluted tubules to be injured (vimentin-positive) and that the percentage of the positive tubules was positively correlated with urinary NAG (uNAG) in all patients, even the non-AKI group of MCNS patients. This suggests that uNAG levels may reflect the degree of subclinical tubular injury in some patient groups [54]. In addition to the diagnosis and prediction of AKI, identification of its etiology is also of importance in different clinical settings, as this provides the possibility of more accurate diagnosis and targeted treatment. This was demonstrated by Kim et al., who aimed to differentiate types of AKI in patients with decompensated liver cirrhosis, namely azotemia, hepatorenal syndrome, and acute tubular necrosis, using uNAG levels, which they found to be a marker specific to renal tubular damage in decompensated cirrhosis, thereby determining the underlying clinical entity and influencing the choice of treatment [55]. Efforts have also been made to evaluate whether combining uNAG, a marker of tubular injury, with existing markers of functional kidney damage improves models of AKI prediction in different patient groups. Ma et al. found that uNAG may be used in combination with serum cystatin C (sCysC), which reflects functional kidney damage, to predict AKI in septic patients [48]. The same combination has also been found to improve the predictive accuracy of AKI in post-operative neurosurgical patients. Similar results were obtained in a different multicentre study of critically ill patients [49]. Additionally, uNAG in combination with sCysC succeeded in improving the accuracy of AKI detection models and intensive care unit mortality prediction [56].

Its applicability as a marker of AKI has been studied in various age groups, which is illustrated by several studies, including the study of Bíró et al., which showed serial uNAG tests (at least 5 samples/patient) in a group of pediatric patients with neoplastic disorders to identify 1.5× more clinical and subclinical AKI episodes, with a relatively high sensitivity and specificity. They also highlighted that serial uNAG measurements decrease the number of false positives, which they mostly attributed to overhydration [57]. Increased levels of uNAG were also found in a study group of pediatric patients with AKI. In the latter study, in addition to a diagnosis of AKI, uNAG was also an indicator of dialysis requirement [58].

In order to establish uNAG’s predictive value for AKI in clinical use, the dependence of its levels on other parameters has to be evaluated, as well as its performance in acutely or critically ill patients. Changes in levels of thyroid hormones and blood glucose have been shown to influence uNAG levels [59,60]. However, targeted studies showed that this did not affect uNAG’s ability to detect AKI. Namely, the results of Wang et al. and Liang et al. showed that blood glucose and HbA1c levels, as well as thyroid hormone levels, did not significantly affect the performance of uNAG for AKI detection [61,62].

The existing body of research shows promise for uNAG’s role as an easily accessible marker for early detection, and even prediction, of AKI. These are crucial for treatment initiation, thereby facilitating better outcomes and, in some cases, prevention of CKD. However, the use of uNAG in AKI also has some limitations. Among others, as in all urinary sampling, obtaining adequate samples from oliguric or anuric patients presents a substantial challenge; conversely, hydration levels also seem to cause variations in uNAG levels, thus blurring the line of clinical significance. Additionally, a possible limitation of NAG is the fact that urinary excretion of the enzyme is elevated not only in acute but also in chronic glomerular diseases [45].

### 3.2. NAG in the Setting of CKD

CKD is a heterogeneous disease that may occur due to multiple underlying disorders and is defined by the KDIGO initiative as “abnormalities of kidney structure and/or function, present for over three months, with implications for health” [63,64,65]. Causes of the disease can vary from genetic heritability to diabetes, hypertension, and glomerulonephritis as well as heart disease, obesity, and old age [66,67]. CKD is a major health problem, affecting over 10% of the world’s population, and is commonly stratified based on the glomerular filtration rate (GFR) and albuminuria [63,68,69]. GFR is considered to be the most important marker of kidney function; however, it is usually not directly measured but rather estimated (eGFR) based on equations employing the serum concentration of creatinine [70]. Combining eGFR with microalbuminuria increases the ability to predict progression toward end-stage renal disease (ESRD) [71,72]. However, due to the heterogeneity of CKD, new and precise biomarkers are needed in order to detect patients in whom interventions are needed in order to halt CKD progression. Furthermore, the clinical need for biomarkers of CKD progression is especially emphasized in patients with early disease; specifically, in whom eGFR exceeds 60 mL/min/m^2^ [73,74]. Therefore, potential biomarkers of CKD are constantly being researched, often using various “omics” approaches, of which proteomics is the most prominent [75,76,77].

uNAG is a potential biomarker of early CKD. Several cross-sectional studies observed higher levels of uNAG in patients with CKD in comparison to healthy controls. Furthermore, in a nested case-control study performed by Kern et al., urinary NAG concentrations measured at the study baseline were successful predictors of micro- and macroalbuminuria in patients with type I diabetes mellitus. Additionally, Vaidya et al. found that lower uNAG levels were associated with regression of microalbuminuria in patients with type I diabetes mellitus [78,79]. However, prospective studies conducted by Lobato et al. and Hsu et al. did not find uNAG to be independently associated with CKD progression [74,80]. We present a selected overview of studies pertaining to NAG as a potential biomarker of CKD progression in Table 1. Even though no association was found for uNAG, Lobato et al. found that NGAL and KIM-1 do seem to be good predictors of CKD progression [80]. In a prospective study conducted by Fufaa et al. on Pima Indians with type II diabetes mellitus, NGAL was significantly associated with ESRD and mortality, which was not observed for NAG [81]. Interestingly, in a study conducted by Hsu et al., neither of the tested tubular damage markers (NGAL, KIM-1, or NAG) did not improve the C-statistic of the baseline clinical prediction model for CKD progression, which employed eGFR and urine albumin to creatinine ratio [73,74]. Based on the available studies, it is clear that uNAG is not an optimal biomarker for CKD progression.

### 3.3. NAG in Kidney Injury Related to Environmental Nephrotoxins

Besides the common intrinsic acute/chronic kidney disease risk factors, several significant environmental influences have been described. The high blood flow rate makes the kidneys susceptible to nephrotoxic substances, which are widely distributed in the environment. Tobacco smoking, environmental pollutants, and occupational-linked toxins such as metals, aristolochic acid, mycotoxins, and solvents alike, are major contributors to this public health issue [86].

Cadmium (Cd) is one of the most toxic heavy metals used in several industrial fields and it is mainly derived from pigments, nickel-cadmium batteries, metal coatings, etc. People are usually exposed to Cd via food, water, children’s plastic toys, air, and tobacco smoking [87]. Cadmium has a long biological half-life and an affinity to accumulate in several organs, especially in the kidneys, which can cause tissue damage and the development of kidney disease, causing polyuria and proteinuria. The proximal tubule is the major site of chronic Cd exposure/deposition, leading to “cadmium nephropathy”, proximal renal tubular dysfunction characterized by epithelial cell hypertrophy, polyuria, and proteinuria [87,88,89]. The standard method used to assess exposure is Cd excreted in urine (U-Cd). In people exposed to low levels of Cd, Akerstrom et al. showed a clear positive association between its urinary excretion and the excretion of urinary proteins. Both low- and high-molecular-weight biomarkers of impaired kidney function were found (U-albumin; U-alpha-1-macroglobulin). However, no direct toxic effect of Cd on kidney function was observed, and the association was explained as a change in urinary flow rate influencing the excretion of both Cd and proteins [90]. In order to estimate the benchmark dose of Cd on kidney function, a random effect meta-analysis was used. U-Cd was correlated to NAG levels, and it was found that age, but not gender, significantly affected the benchmark dose estimation [91]. A study of urine samples from 185 non-smoking female farmers from two rural areas in southern China showed that NAG is a better biomarker of long-term environmental Cd exposure than eight other tentative biomarkers [89]. The toxic effect of cadmium on renal function is still to be determined, due to the significant variability in Cd exposure and the initial health of the studied patients (early or late-stage kidney damage).

**Figure 2:**
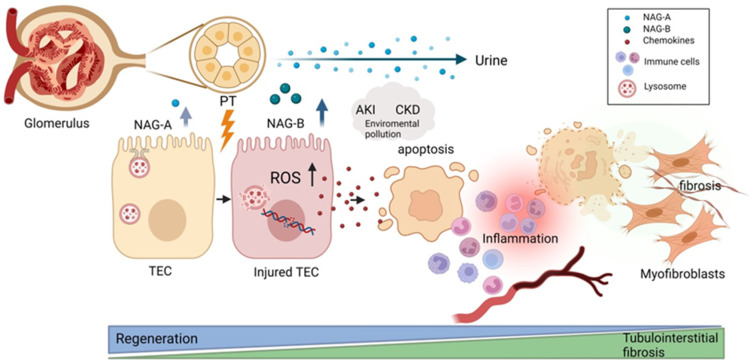
Schematic illustration of NAG excretion to urine upon kidney injury in relation to regenerative vs. fibrotic kidney processes. Kidney stress induces the activation of the ROS pathway, triggering the apoptosis/necrosis of proximal tubular epithelial cells (PTECs). If the sustained injury overwhelms the regenerative capacity of the tissue, the proinflammatory and profibrotic cascade can take over. The released chemokines then support the infiltration of immune cells, resulting in acute kidney injury, which can over time progress to chronic kidney disease. NAG-B isoform is readily excreted to urine after kidney injury as a result of lysosomal damage in comparison to NAG-A, which is normally found in small amounts in healthy urine samples. PTEC—proximal tubular epithelial cell; NAG—N-acetyl-beta-D-glucosaminidase; PT—proximal tubule; AKI—acute kidney injury; CKD—chronic kidney disease; ROS—reactive oxygen species. Image created with Biorender.com.

The innate mechanism to compensate for the toxic effects of cadmium accumulation (and other metals) is mediated by highly conserved, cystein-rich metal binding proteins called metallothioneins, that form cadmium-metallothionein complex [87]. In high chronic exposure situations, these proteins are depleted and the nephrotoxic effect of cadmium is triggered by oxidative stress and the release of reactive oxygen species [92] (Figure 2). This causes DNA damage, protein oxidation, and lipid peroxidation, which in turn leads to tissue necrosis and apoptosis. The gastrointestinal absorption of Cd is affected by body levels of iron (Fe) and zinc (Zn), and Cd also interacts with some metals, such as Zn, copper (Cu), calcium (Ca), and Fe. As these metals compete for metallothioneins, elevated bodily Cd levels lead to a Cu to Zn ratio (CZR) imbalance, which is an additional risk factor for renal damage that is independent of Cd. Interestingly, the uNAG activity was associated with U-Cd but not with the CZR, suggesting a different molecular mechanism leading to kidney damage [93]. Alongside Cd, exposure to other nephrotoxic metals from drinking water is a widespread concern. Some epidemiologic studies suggest that elevated exposure to arsenic is associated with CKD, although the mechanism seems unrelated to NAG [94,95]. A recent systematic review confirmed a positive correlation between Cd exposure and NAG levels and also elevated arsenic and chromium exposure in relationship to KIM-1 [96].

Other sources of nephrotoxic compounds are microfungi that naturally produce over 500 mycotoxins that can be present in one’s diet and have an impact on human health. Over years, oral exposure to mycotoxins can induce renal damage, which was confirmed in a number of studies that correlated Ochratoxin A (OTA), Citrinin (CIT), Zearalenone, Fumonisin B1, Sterigmatocystin, and Alfatoxin B with nephron segment injury, predominantly proximal tubule damage [97,98]. The use of uNAG was suggested as an early marker of kidney damage induced by OTA and CIT [99].

Kidney injury can often be drug-induced and is common (up to 37.5%) in patients in intensive care units [100]. The drug dose-limiting factor is usually related to the damage suffered by the PTECs that mediate inflammation in the early stages of kidney disease. Activated PTECs secrete chemokines, which have an important role in the recruitment of immune cells into the kidney interstitium and inhibit autologous immune responses [101]. An in-depth approach to assess the PTECs’ cellular damage, called Nephroscreen, was recently developed. It uses a microfluidic platform consisting of multiple chips used to assess various toxicologically relevant parameters, including NAG, to estimate membrane integrity. After extensive evaluation of the sensitivity and specificity, such a platform has the potential to be used for drug toxicity assays and to estimate drug-drug interactions [102].

Multiple environmental and intrinsic factors can significantly induce kidney injury, which usually results from damage induced to PTECs. Among other biomarkers in use, uNAG helps assess the mentioned damage, specifically, without going into any details about the molecular aspects of its occurrence in urine. Therefore, more studies should be carried out in order to identify the turning point in a series of events leading to damage of the kidney segments and later connect it to the most reliable biomarker.

## 4. Identifying Potential Drug Targets in Kidney-Disease-Associated Signaling Pathways

The injury of PTECs triggers multiple pathways that contribute to kidney damage. The activated oxidative stress pathway causes an imbalance between the reactive oxygen species production and the body’s antioxidant defenses, in turn damaging proteins, lipids, and DNA and causing cell death mediated by caspases and/or endonucleases [103]. Governed by the activation of the nuclear factor-κB pathway, the damaged PTECs release pro-inflammatory cytokines and chemokines, recruiting immune cells, sustaining the inflammatory response, and contributing to the development of CKD [104]. The severe or prolonged stress in PTECs can also trigger caspase-mediated apoptosis through the endoplasmic reticulum stress pathway [105]. In such advanced disease stages, the onset of renal interstitial fibrosis is typical, promoted by the Wnt/*β*-catenin signaling pathway [106]. Although significant progress was made to identify the renal signalling pathways activated in acute injury, the mechanisms contributing to the transition to CKD are unclear [107]. The potential role of NAG in these processes deserves additional study, as targeting the key factors and pathways may provide the much-needed therapeutic strategies for preventing or slowing the progression of CKD [108].

In order to enhance the validity of the above findings, advanced technologies can be applied to provide additional insight. Computational drug target identification and drug discovery approaches can accelerate drug development and improve patient outcomes. Network-based analysis and structural biology can help identify key molecular pathways and, within them, specific nodes in the protein interaction networks that are likely to be critical in kidney disease pathogenesis. Aided by machine learning algorithms that analyze large datasets and identify complex patterns, the study of the three-dimensional structure of key proteins can uncover potential drug-binding sites and further the design of customized drugs [11]. Finally, with the use of genomics and transcriptomics data, the genetic variants and gene expression patterns associated with kidney diseases can be identified, paving the way to the development of more personalized therapies [109].

## 5. Conclusions

As the renal tissue is stressed, the underlying inflammatory and profibrotic molecular pathways mediate acute and chronic kidney damage. Currently, there is no “golden standard” for the diagnosis of early, subclinical kidney disease, and it is therefore difficult to conduct a validation test of uNAG (or other biomarkers) in the setting of early disease onset. In this review, we present an overview of research pertaining to uNAG’s potential to fill this niche. Even though a large body of evidence exists suggesting a clear connection between uNAG and kidney injury, further cohort studies are needed focusing on the kinetics of uNAG in different etiologies of kidney disease. Its possible role as a diagnostic molecule is supported by the fact that in physiologic conditions its urinary levels are insignificant; however, as the renal tubule epithelium becomes permeable under stress, its urinary levels increase sharply [22]. Although this rise is deemed to be a more sensitive kidney damage indicator than creatinine, crucially, the raise in uNAG expression seems to be unspecific of the underlying kidney disease [35]. Given its sensitivity, uNAG is unsurprizingly a well-established target that has been extensively studied in multiple settings related to clinical and subclinical kidney injury, nephrotoxic substances, and secondary conditions such as cardiovascular disease and liver cirrhosis and in septic, post-operative or critically ill patients [48,49,52,53,54,55,56]. Additionally, in CKD it has shown promise in early detection; however, it failed to detect signs of disease progression [74,80]. uNAG was also not succesful in differentiating between acute and chronic kidney disorders, which is, in part, due to the fact that they can be caused by multiple underlying noxious stimuli. Furthermore, these diseases are a continuum, and it is not always possible to clearly distinguish between the two clinical entities. Considering the high sensitivity of uNAG to stress but the apparent lack of specificity, its role as a unique biomarker of a certain kidney-related condition is therefore unlikely. However, as multiple studies have suggested, it could be useful as part of a biomarker panel, in which uNAG is utilized as an early general stress indicator, while other potential biomarkers, such as NGAL, Kim-1, or sCysC, could convey the needed disease specificity [35,48,49,56].

Finally, whether NAG could be used as a potential drug target in kidney disease remains to be seen. Machine learning methods could be employed to predict drug–target interactions and a novel computational model could be developed to identify drug–target interactions based on the protein sequence, structure, and drug chemicals [110]. Moreover, protein–protein interactions involving uNAG should be analyzed to further clarify its potential as a target molecule in kidney indications [111,112].

## Figures and Tables

**Table 1:** Selected overview of the research regarding urinary N-acetyl-β-D-glucosaminidase (uNAG) as a potential predictor of CKD progression.

Authors, Year	Type of Study	Conclusions
Holdt-Lehmann et al., 2000 [82]	Cross-sectional study	uNAG was significantly elevated in patients with glomerulonephritis compared with healthy controls.
Bazzi et al., 2002 [83]	Cross-sectional study	uNAG excretion can be considered as a reliable marker of the tubulo-toxicity of proteinuria in the early stage of IMN, FSGS, and MCD.
Kern et al., 2010 [78]	Nested case-control during 1-9 years of follow-up	Elevated uNAG at baseline independently predicted micro- and macroalbuminuria in patients with type I diabetes metllitus.
Vaidya et al., 2011 [79]	Prospective study during 2 years of follow-up	Lower uNAG levels were associated with regression of microalbuminuria in patients with type I diabetes mellitus.
Kim et al., 2016 [43]	Cross-sectional study	uNAG may be related to glycemic parameters reflecting glucose fluctuation and decreased insulin secretory capacity in patients with type II diabetes mellitus.
Jungbauer et al., 2016 [84]	Prospective study during 5-year follow-up	Elevated uNAG was an independent predictor of ESRD and all-cause mortality in HF patients.
Lobato et al., 2017 [80]	Prospective study during median 15-months follow-up	uNAG levels were weakly correlated with CKD stages I-V defined by KDIGO; uNAG was NOT associated with CKD progression or renal adverse outcomes.
Hsu et al., 2017 [74]	Prospective study (9433 person-years)	uNAG was associated with CKD progression before adjustment. After adjustment for established risk factors of CKD, there was no independent association.
An et al., 2019 [85]	Cross-sectional study	uNAG was increased in patients with IMN, compared with healthy controls. No correlation between histological grade and uNAG was observed.

FSGS—focal segmental glomerulosclerosis; HF—congestive heart failure; IMN—idiopathic membranous nephropathy; MCD—minimal change disease; uNAG—urinary N-acetyl-β-D-glucosaminidase.

## Data Availability

No new data were created or analyzed in this study. Data sharing is not applicable to this article.
